# Validation and Analysis of the European Quality Questionnaire in Italian Language

**DOI:** 10.3390/ijerph17238852

**Published:** 2020-11-28

**Authors:** Leopoldo M. Amendola, Alessandro Galazzi, Irene Zainaghi, Ivan Cortinovis, Anna Zolin, Rik T. Gerritsen, Ileana Adamini, Maura Lusignani, Dario Laquintana

**Affiliations:** 1 Direction of Healthcare Professions, Foundation IRCCS Ca’ Granda Ospedale Maggiore Policlinico, Via Francesco Sforza 35, 20122 Milan, Italy; leopoldo.amendola@policlinico.mi.it (L.M.A.); irene.zainaghi@policlinico.mi.it (I.Z.); ileana.adamini@policlinico.mi.it (I.A.); dario.laquintana@policlinico.mi.it (D.L.); 2 Department of Sciences for Health, University of Florence, Piazza San Marco 4, 50121 Florence, Italy; 3 Laboratory G.A Maccacaro, Department of Clinical Sciences and Community Health, University of Milan, Via Vanzetti 5, 20133 Milan, Italy; ivan.cortinovis@unimi.it (I.C.); anna.zolin@unimi.it (A.Z.); 4 Department of Intensive Care, Medical Center Leeuwarden, Henri Dunantweg 2, 8934 Leeuwarden, The Netherlands; RTGerritsen@ZNB.nl; 5 Department of Biomedical Sciences for Health, University of Milan, Via Pascal 36, 20133 Milan, Italy; maura.lusignani@unimi.it

**Keywords:** communication, EuroQ2, family, FS-ICU, Intensive Care Unit, QODD, satisfaction, validation study

## Abstract

The European Quality Questionnaire (euroQ2) is the culturally-adapted version to the European context of the Family Satisfaction in Intensive Care Unit (FS-ICU) and Quality of Dying and Death (QODD) tools in a single instrument divided into three parts (the last is optional). These tools were created for an adult setting. The aim of this study was the Italian validation and analysis of the euroQ2 tool. The Italian version of euroQ2 questionnaire was administered to the relatives, over 18 years of age, of adult intensive care unit patients, with the Hospital Anxiety and Depression Scale (HADS) and the Impact of Event Scale—Revised (IES-r). For the re-test phase the questionnaire was administered a second time. One hundred questionnaires were filled in. The agreement between test and retest was between 17–19 out of 20 participants with an upward trend in the re-test phase. A measure of coherence and cohesion between the euroQ2 variables was given by Cronbach’s alpha: in the first part of the questionnaire alpha was 0.82, in the second part it was 0.89. The linear Pearson’s correlation coefficients between all questions showed a weak positive correlation. The results obtained agreed with the original study. This study showed a good stability of the answers, an indication of an unambiguous understanding of the Italian translation.

## 1. Introduction

The literature points to Family Satisfaction in the Intensive Care Unit (ICU) as one of the main end-points to be analyzed in order to assess clinical treatment and communication and to implement actions to improve them [1,2].

When a relative is admitted to the ICU, it is not easy to understand the information and the situation itself. This difficulty generates in the family members a sense of impotence, stress, anxiety and post-traumatic stress syndrome [3]. Therefore, the communicative relationship in the ICU is an extremely complex element in terms of satisfaction and qualitative evaluation.

The information should be given verbally, in accessible language: explained, therefore, in simple words, by repeated messages [1,2,4]. Effective and well-structured communication aims, on the one hand, to facilitate the clinical-assistance staff on the patient’s journey and, on the other hand, to meet the needs of family members, such as building trust, providing emotional support, providing medical information, enabling understanding of the patient as a person and facilitating joint therapeutic decisions [1,5,6,7].

In order to improve relational and communicative aspects, however, there is a need to have new professional figures and tools for the evaluation of these aspects [8,9,10,11,12,13,14,15]. The literature offers numerous examples of tools to measure and evaluate family member satisfaction. Among these, for adult patients, are: the Critical Care Family Needs Inventory (CCFNI), the Society of Critical Care Medicine Family Needs Assessment (SCCMFNA), the Critical Care Family Satisfaction Survey (CCFSS) and the Family Satisfaction in the Intensive Care Unit (FS-ICU) [16,17,18,19,20,21,22]. For pediatric patients the Society of Critical Care Medicine Family Needs Assessment (SCCMFNA) has been used, although no instruments in this category have been developed specifically for pediatric ICU settings [16].

In 2015, the euroFS-ICU (European version of the FS-ICU) was born, and in association with the Quality of Dying and Death (QODD), it became the backbone of the euroQ2 project, aimed at assessing the satisfaction of family members of patients in ICU in Europe [23,24]. The two original instruments (FS-ICU and QODD) are among the most used, tested, analyzed and reviewed tools in the literature [16]. In addition, where both instruments have been used, a strong correlation of scores related to satisfaction with treatment could be observed, although QODD was not specifically created for the ICU [16].

The euroQ2 questionnaire is the culturally adapted version of the FS-ICU and QODD instruments gathered in a single instrument divided into 3 parts [23,24]. Like its predecessors, euroQ2 is a versatile tool, useful for the evaluation of quality indicators in the clinical governance process [20,21,22,23,24]. The versatility and reliability of the original instruments has therefore been adapted to the European context in a simpler and clearer single format [25,26]. Moreover, it has been successfully used both to highlight possible areas of improvement within the unit and to evaluate the effectiveness of training activities [20,21,22,23,24].

It has not been possible to find a quality assessment tool in the ICU that has been validated in Italy. Since euroQ2 is a derivative questionnaire, adapted to the European reality starting from widely tested instruments, recently built and currently under study in nine countries of the European Community, it was decided, therefore, to perform the validation of this instrument. The aim of this study was the Italian validation and analysis of the euroQ2 tool.

## 2. Materials and Methods

### 2.1. Instrument Description

The euroQ2 questionnaire consists of an introductory part that collects the personal data of those who fill it in: age, gender, and degree of relationship to the patient. It then consists of three evaluation areas (see Supplementary Materials A): Treatment (part 1), Communication (part 2), and End-of-Life Treatment (part 3—optional).

### 2.2. Setting

The study was conducted at the General ICU (12 beds) and the Neuroscience ICU (6 beds) of the Fondazione IRCCS Ca’ Granda Ospedale Maggiore Policlinico, an academic tertiary-level hospital in Milan.

### 2.3. Study Design

A monocentric validation study was conducted. The original text of the instrument [23,24], published in English, has been translated and adapted (forward translation) to the Italian language and culture (see Supplementary Materials A). The maintenance of the original contents was verified through back-translation by two English native speakers. The original authors of euroQ2 endorsed the study.

The questionnaire was subjected to pre-testing and expert examination, and administered in the initial phase to 12 nurses and 6 doctors of the ICUs, in order to ascertain the clarity and comprehensibility of the questions and to check if there were terms or concepts to be explored in more detail or more simply. The questionnaire was considered clear and concise in its form and there was no need for further explanation or clarification for the ICU staff.

The euroQ2 was translated into Italian and proposed to the relatives of the patients in ICU with the Hospital Anxiety and Depression Scale (HADS) [23] and Impact of Event Scale—Revised (IES-r) [24]. As in the original study [25,26] HADS and IES-r, were used to assess the possible association between the responses to euroQ2 and the emotional state of the relatives themselves. The euroQ2 was administered close to the time when the patient was transferred to the wards.

A Re-test phase was carried out by administering the euroQ2 questionnaire a second time to family members who had joined the study. In order to estimate a 95% concordance between the two answers obtained from the same subjects with a lower limit of the confidence interval of 85%, 20 family members were necessary. The Re-test of the questionnaire took place from four days to one week after the patient discharge from the ICU and the first administration of the questionnaire. The 20 relatives recruited for this phase of the project joined voluntarily. The Re-test took place maintaining the anonymity of the participants as in the previous phases of the study, coupling by code the original questionnaire to the Re-test questionnaire after the compilation. The phases of the study are in accordance with the Process of Translation and Adaptation of Instrument of the World Health Organization [27].

### 2.4. Study Population

The questionnaires were collected consecutively between April and June 2019. The population was represented by 100 family members of adult ICU patients who had been in the ICU for more than 48 h. The questionnaire was administered anonymously.

### 2.5. Inclusion and Exclusion Criteria

Family members over 18 years of age of both sexes who signed informed consent were included in the study population. Only one family member per hospitalized patient was recruited.

Family members unable to understand the Italian language were excluded.

### 2.6. Statistical Analysis

The data collected were described for each of the variables of the euroQ2, anxiety and depression scores obtained by the HADS subscales, and the attitudes described by the IES-r subscales. The descriptions were also made according to age, gender and parental relationship with the patient.

The chi-square test was used to evaluate the association between the variables considered and Cronbach’s alpha was used to detect the degree of cohesion and homogeneity in the answers for questions of the same topic [28]. To evaluate the concordance of the results in the Re-test, Cohen’s Kappa was used [29]. For all the tests, the statistical significance was evaluated with a threshold of 0.05 (first type error). In order to describe the relationships between the different answers to the euroQ2 and to verify if the domains present in the original work were also found in the present study, the multiple correspondence analysis (MCA) and cluster analysis (by Ward algorithm) were used. Four clusters were obtained; the description of the answers of each cluster shows the profile of four groups of relatives. For more details on statistical analysis see Supplementary Materials B.

### 2.7. Ethical Considerations

The study was authorized by the Milan Area 2 Ethics Committee on 02/04/2019 number 941.

## 3. Results

### 3.1. Patient and Relative Description

One hundred questionnaires were distributed and filled in by relatives of ICU patients. Sixty-three percent of these patients were male, 50% of patients were aged 60 years or over and 46% had urgent surgical problems. The median length of ICU stay was about 4 days (IQR 3-8) and a median intubation period of 2 days (IQR 1-4).

Thirty-one percent of the family members participating in the study were male, 57% were aged between 40 and 59 years, 33% were partners of the patient, 35% were sons or daughters, and 16% were parents (Table 1).

Table 2 and Table 3 show the description of the answers obtained from the two questionnaires HADS and IES-r, used to evaluate the emotional involvement and psychological mechanisms of the relative towards the patient’s situation. The only statistically significant associations (*p* < 0.05) were found between relatives and depression and between age and avoidance attitude.

### 3.2. Re-Test Analysis

Coincident or at most different answers of step 1 were considered “agreement”: for example, if the first answer is “good”, the second could be “very good” or “sufficient”.

The minimum concordance obtained in the retest was 17 out of 20 participants to retest trial (all the 17 answers agreed for test and retest). However, the agreement between test and retest was in most questions, between 17–19 out of 20 with a tendency to have a more positive assessment in the second test.

The concordance between the test and retest with Cohen’s K values is between 0.503 and 0.8897 except for a single value attested to 0.4521 (item 2 part 1 of the questionnaire: Symptom management—Pain).

The percentage of maximum difference obtained between the two answers to the same question (greater than step 1) was 15% with a tendency to a more positive assessment than the test. The items most affected by this trend are those that investigated the ICU environment and the quality of the support provided by the staff.

### 3.3. Validation of the Questionnaire

**Figure 1:**
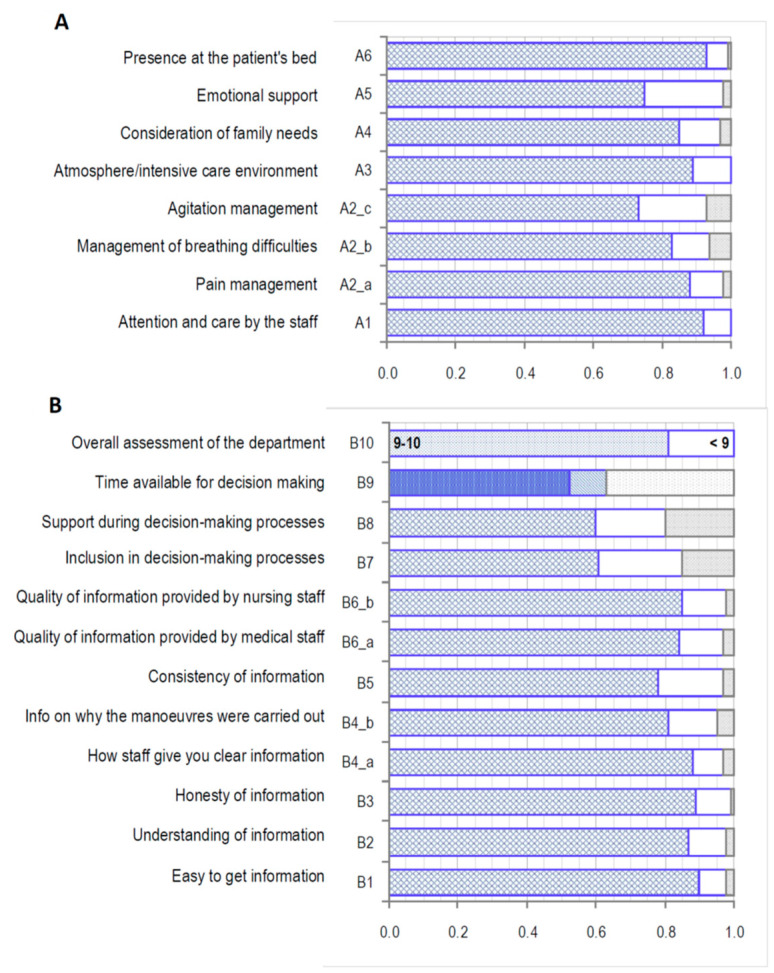
Answers obtained to each question of the questionnaire. (**A**) first part of the questionnaire; (**B**) second part of the questionnaire.

The description of the answers to all the questions is given in Figure 1. In the first part of the questionnaire (Figure 1A), at least 80% of the answers are “excellent” or “very good”. In the second part instead (Figure 1B), the best (“excellent” and “very good”) answers reach only 60% in the questions about support or inclusion in decision-making processes (B7 and B8), the remaining answers are all above 75%.

A measure of coherence and cohesion between the euroQ2 questions is given by Cronbach’s alpha. For the first part of the questionnaire the alpha is 0.82. In the first part of the questionnaire, excluding from the computation of the alpha one question at a time, there are no important variations (from 0.77 to 0.83).

The questions of the second part of the questionnaire seem to be more coherent and cohesive. Cronbach’s alpha is 0.89 and the exclusion of one of the questions at time shows a variation of the alpha between 0.88 and 0.90.

The calculation of linear Pearson’s correlation coefficients between the questions of the questionnaire shows a positive correlation that tends to be weak (only 2 exceed the value 0.7: A4–A3 and B6_a–B5).

None of the answers to the euroQ2 questionnaire was statistically associated with the age of the respondents.

The relationship of the respondents was statistically associated with the questions A4 (considering needs), B3 (honesty in the information provided), and B6 (quality of information provided by nurses).

For the HADS score, both sub-scales (anxiety and depression) as for the IES-r intrusion sub-scale were not associated with the answers to euroQ2.

For the IES-r avoidance sub-scale the only question whose answers are statistically associated is B7 (inclusion in decision-making processes). For the IES-r hyperarousal sub-scale the only associated variable seems to be A1 (care by staff members). As far as the overall evaluation of the IES-r score is concerned, the agitation management seems to be associated (A2_c).

### 3.4. EuroQ2 Analysis Part 3 (Euro-QODD)

It was not possible to carry out a statistical analysis of the data for part 3 of euroQ2 due to the small number of situations investigated during the survey period. In fact, the end-of-life data collected during the study period refer only to 2 patients.

### 3.5. Results and Analysis of Open Answers

Analyzing the open answers, only 45% of the people surveyed provided further evaluations and opinions with respect to the questions already posed by the instrument. Of these, 23% used it to express their gratitude and appreciation for the efficiency, preparation and humanity of the staff.

The area most affected by the open-ended responses was communication. Seven percent of the total participants highlighted the need for greater attention to the relational area, stressing their need for more possibilities to communicate with the medical staff (especially in the morning even only by telephone), with more frequent updates within 24 h, and the importance of having a single referent in the communication area that also allows less relational variability. The family members also highlighted the excessive severity in limiting the number of family members who can receive information from the medical staff (1 family member per patient) and suggested the implementation of a psychological support service dedicated to relatives who request it, in particular cases such as end-of-life treatment. Furthermore, they expressed the need for greater flexibility in visiting hours and in the number of relatives per visit.

Seven percent of the total relatives involved in the study used the open response spaces to highlight critical events in the management of the ICU and care of the patient. These problems, summarized in Table 4, considered as “critical events”, should never have occurred and they were discussed during the ICU monthly meetings with all team members.

### 3.6. Respondent Profiles

In order to evaluate the relationships between the answers to the different questions and to verify whether the domains of the original questionnaire were similar to those obtained with the Italian translation, we used the MCA. This analysis showed that the first two factorial axes explain more than 72% of all the information present in the sample.

The first factorial axis (47.6%) contrasts positive and negative judgments on the aspects of information, pain management, and agitation. In the second axis (24.9%) there is a qualitative element of the information received and in the third axis (8.3%) the judgements on the understanding of the information provided are differentiated, bringing out the role of the nurse as a language mediator.

The only item present in the original euroQ2 study, but not very informative in our sample responses, is “Support during decision making”.

With the cluster analysis we found 4 different response profiles of relatives (see Statistical Analysis Supplementary Materials B). First, sons/daughters or partners of the patient, younger than 60 years, that express a positive judgment on the management of the patient. Second, people with avoidance attitude, over-60s, and anxious, that express a positive judgement on the management of the patient. Third, older people, with little anxiety but a lot of hyperarousal, showing less than others a positive judgement on the management of the patient. Fourth, a high proportion of women, mostly daughters, less than 60 years old, anxious people, showing less than others a positive judgement on the management of the patient.

## 4. Discussion

The results of the statistical analysis indicate adequate coherence and internal cohesion of the instrument comparing to the Danish-Dutch original articles [25,26].

The translated questionnaire showed, therefore, an adequate level of stability and objectivity maintaining a high percentage of concordance between the test and the retest.

The absence of associations among the scores obtained by HADS, IES-r and the almost all variables of euroQ2 are a strong support for the hypothesis of the negligible emotional influence on the given responses. The ICU staff should be aware that symptoms related to post-traumatic stress disorder can occur in the relatives of patients admitted to the ICU. Recognizing these symptoms is essential for a proper communication process with the patient’s family members during an ICU stay, and even more essential in the case of an end-of-life treatment [3].

The retest results indicate a good stability of the instrument (clarity and unambiguity of the translated questions) with an upward trend in the evaluation of the individual items compared to the test. This trend could be related to the comparison of the relatives’ reality of the ICU with that of other departments after discharge. This, of course, is only a hypothesis not supported by the analysis.

The results of linear Pearson’s correlation coefficients may suggest that each of the questions investigates different aspects of the intensive care experience.

The use of the open response to the questionnaire has proved extremely useful to highlight weaknesses in the structure or service and to highlight critical events related to practice, making the study participants an integral part of the analysis and quality improvement process in the unit.

Part 3 of euroQ2 (euro-QODD) was excluded from the statistical analysis due to a small number of deceased patients during the study period. Although the evaluations collected from the participants indicate attention and care in the end-of-life treatment process towards both family members and the patient, it is necessary to underline the difficulty in administering this part of the questionnaire.

Further studies are recommended for this component of the instrument, with particular attention to the timing and methods of administration and taking into account the cultural component shared by most of the family members and by many in the ICU staff who find it difficult to talk about the unfortunate event as a form of respect towards the deceased and his/her family members.

The MCA and clusters analysis shows the great importance of the items related to the quality of communication to the family members in the ICU. Cluster three’s data (low evaluation of the communication between healthcare professionals and relatives) lead us to hypothesize that, since data refer mainly to participants not closely related to the patients, their answers and scores tend to be lower and are linked to the need, according to the privacy law, not to provide information to people not closely related without the patient’s authorization. This obligation often forces the communication action to be delayed or limited with a considerable impact on the overall perceived quality. Of course, this is only an interpretative hypothesis of the results obtained.

The instrument has been widely appreciated by the relatives of the patients who saw the opportunity to express their opinion as an increased interest of the staff towards them and their relative, as well as a sincere desire to continue to improve the service.

The results obtained from the study are comparable and congruent with those presented by the original validation study.

The euroQ2 is also an effective tool for staff training: it highlights the areas where it is necessary to implement training or further education for the whole healthcare team.

### 4.1. Study Limitations

This study has some limitations. First, it was impossible to compare the data obtained from the administration of euroQ2 with other instruments belonging to the same class because, at the moment to our knowledge, there are no other validated questionnaires in Italy, nor is there a gold standard of comparison in the literature. Second, the third part of the questionnaire was not analyzed. It is considered appropriate and advisable to continue with further studies on this instrument.

### 4.2. Future Research Directions

The validation of the euroQ2 in Italian allows it to be used by Italian native speakers and the analyses carried out reinforce what has already been described by the creators.

All ICU staff members involved in the process of analyzing the quality of communication and clinical practice could benefit from this tool.

The key elements that could be drawn from the euroQ2 are: the possibility of improving clinical practice and training with the help of the service users themselves, the possibility to improve the organization of the service itself for the benefit of users and staff, and, finally, the possibility to make users an effective resource to the overall qualitative growth of the service, making them, at the same time, aware of the actions carried out, involved in the decision-making process and with increased knowledge of the event that involved their process of care and assistance jointly undertaken.

## 5. Conclusions

In the ICU, with the shift towards a patient-centered perspective and an open visitation policy, it is vital to analyze user satisfaction data. The family members, faced with the impossibility, in many cases, of the patients to express their opinion, become the focus of this research on satisfaction.

The data collected is effective in evaluating the activity of the unit, the organization of the operational unit and the activity of the staff, especially in the communication field.

The tested and validated tool proved to be stable, consistent and objective. EuroQ2 was appreciated by family members who saw it as an opportunity to express their feelings and opinions, a form of interest in them and a genuine desire of the staff to improve and project themselves towards a service always aimed at excellence. The feedback provided by the patients’ relatives proved extremely useful to highlight critical events, making them de facto participants in the actual improvement of the quality of service.

## Figures and Tables

**Table 1:** Characteristics of the interviewees.

Variable	Characteristics	% (*n* = 100)
Age	<40 years	22
	40–59 years	57
	≥60 years	21
Sex	female	69
	male	31
Family members	partner	33
	son/daughter	35
	parent	16
	other	16

**Table 2:** Emotional and stressful condition of the interviewees (HADS questionnaire).

Hospital Anxiety and Depression Scale (HADS)	Median	Q1–Q3	% Patients with Score ≤ 7 (Non Cases)	% Patients with Score > 11 (Cases)
Total score Measure of emotional stress	29 (min = 0; max = 42)	26–30		
Anxiety (Sum of score of 7 questions)	10 (min = 0; max = 21)	8–13	17	39
Depression (Sum of score of 7 questions)	11 (min = 0; max = 21)	9–13	8	36

**Table 3:** Emotional and stressful condition of the interviewees (IES-r questionnaire).

Impact of Event Scale—Revised (r)	Median	Q1–Q3	% Patients with Low Score	% Patients with High Score
Intrusion (Mean of score of 8 questions)	2.0 (min = 0; max = 4)	1.4–2.9	52 *	48 *
Avoidance (Mean of score of 8 questions)	1.1 (min = 0; max = 4)	0.7–1.8	85 *	15 *
Hyperarousal (Mean of score of 6 questions)	1.5 (min = 0; max = 4)	0.7–2.3	70 *	30 *
Total means IES-r score	4.9 (min = 0; max = 12)	3.1–6.8	25 **	55 **

* mean value of subscales: low score ≤ 2; high score >2. ** total score: low score <24; high score ≥ 33 (likely presence of post-traumatic stress disorder). The four main symptoms that define post-traumatic stress disorder are: intrusion, avoidance, negative symptoms, and hyperarousal. Intrusion: the inability to keep memories of the event from returning. Avoidance: an attempt to avoid stimuli and triggers that may bring back those memories. Negative symptoms: ongoing negative feelings about oneself or others, and which may include anger, guilt, and shame, or a decreased ability to experience positive emotions. Hyperarousal: similar to jumpiness, it may include insomnia, a tendency to be easily startled, a constant feeling that danger or disaster is nearby, an inability to concentrate, extreme irritability, or even violent behavior.

**Table 4:** Critical event percentage.

Critical Event	Percentage
The lack of a chair for the relative next to the patient’s bed.	1%
The division of the patient’s personal belongings between the custody in the Emergency Room and in the Intensive Care Unit.	1%
The absence of a meeting with the surgeon who operated on the patient.	1%
The transfer of a patient to the ward without notifying the family members.	1%
The recruitment of a patient in a medical practice without prior communication to family members.	1%
The lack of clinical communication with a family member, even at the explicit request of the patient of for a meeting with medical team failure to open doors during visiting hours to a family member.	1%
The involved relatives suggested environmental improvements such as the addition of vending machines in the waiting room and a better separation (especially acoustically) of the hospital spaces.	2%
The involved relatives said they had difficulty in giving opinions due to their lack of experience in the field and had no terms of comparison.	2%
The relatives declared their amazement at the fact that there was no need to use a mask and over-shoes.	1%
The relatives said they were bothered by the medical staff’s request for information about the dosage of medicines taken at home by the patient.	2%

## Supplementary Materials

The following are available online at https://www.mdpi.com/1660-4601/17/23/8852/s1, Supplementary material A: The Italian euroQ2, Supplementary material B: Statistical analysis of Italian euroQ2.
