# Supplementary material for: Validation and Analysis of the European Quality Questionnaire in Italian Language
Source: Int J Environ Res Public Health. 2020 Nov 28;17(23):8852. doi: 10.3390/ijerph17238852 (PMC7729862; doi:10.3390/ijerph17238852)
Supplement: Supplementary file 1 [file ijerph-17-08852-s001.zip › Supplementary material A Italian euroQ2.pdf]

Supplementary material A:

European Quality Questionnaire (euroQ2) in lingua italiana

Il seguente **questionario** riguarda la **vostra esperienza in terapia intensiva**.
Siamo interessati a conoscere la vostra opinione riguardo al trattamento che avete ricevuto voi (familiari e parenti) e il vostro congiunto, ricoverato presso questa unità operativa, per comprendere la qualità delle cure e della comunicazione clinica, migliorando dove possibile il processo di cura.

All'interno del questionario troverete il termine "membri dello staff di terapia intensiva": questo termine identifica medici, infermieri e tutto il personale dell'unità operativa.

Se desiderate aggiungere precisazioni o ulteriori informazioni in merito alle risposte fornite, potete farlo nei riquadri di commento al termine di ogni parte del questionario.

Se doveste ritenere alcune domande di difficile comprensione o non rilevanti, vi invitiamo a segnalarlo nei riquadri di commento: i vostri suggerimenti saranno tenuti in considerazione.

Il questionario è in **forma anonima**.

Vi **ringraziamo** anticipatamente per la collaborazione.

DATI PERSONALI

In questo spazio vi chiediamo di inserire pochi dati personali.

1. Età………

2. Sesso

Maschio	☐
Femmina	☐

3. Grado di parentela rispetto al paziente

Marito/Moglie o convivente	☐
Figlio/Figlia	☐
Fratello/Sorella	☐
Padre/Madre	☐
Amico/Amica	☐

Altro (si prega di specificare) ________________________________

PARTE 1: SODDISFAZIONE DELLE CURE

In questa sezione, vorremmo porvi alcune domande sulla vostra esperienza complessiva rispetto all'assistenza fornita al vostro familiare (il paziente) e a voi.

Si prega di selezionare la casella che meglio riflette le vostre opinioni. Se la domanda non si applica al ricovero del vostro familiare, selezionare la casella "non applicabile" (N/A).

COME È STATO TRATTATO IL VOSTRO CONGIUNTO (IL PAZIENTE)?

1. **Attenzione e cura da parte dei membri dello staff di terapia intensiva:** cortesia, rispetto e compassione dimostrati nei confronti del vostro congiunto (il paziente)

Eccellente	☐
Molto buona	☐
Buona	☐
Sufficiente	☐
Scarsa	☐
N/A	☐

2. **Gestione dei sintomi:** capacità dei membri dello staff di terapia intensiva di riconoscere e trattare i sintomi manifestati dal vostro congiunto (il paziente)

2 a. Dolore

Eccellente	☐
Molto buona	☐
Buona	☐
Sufficiente	☐
Scarsa	☐
N/A	☐

2 b. Difficoltà respiratoria

Eccellente	☐
Molto buona	☐
Buona	☐
Sufficiente	☐
Scarsa	☐
N/A	☐

2 c. Agitazione

Eccellente	☐
Molto buona	☐
Buona	☐
Sufficiente	☐
Scarsa	☐
N/A	☐

COME SIETE STATI TRATTATI?

3. Ambiente/atmosfera della terapia intensiva: quanto i membri dello staff della terapia intensiva vi hanno fatto sentire apprezzati per la vostra presenza

Eccellente	☐
Molto buona	☐
Buona	☐
Sufficiente	☐
Scarsa	☐
N/A	☐

4. Considerazione dei vostri bisogni: quanto i membri dello staff della terapia intensiva hanno dimostrato interesse nei confronti delle vostre necessità

Eccellente	☐
Molto buona	☐
Buona	☐
Sufficiente	☐
Scarsa	☐
N/A	☐

5. Supporto emotivo: quanto i membri dello staff della terapia intensiva hanno provveduto a darvi il necessario supporto emotivo

Eccellente	☐
Molto buona	☐
Buona	☐
Sufficiente	☐
Scarsa	☐
N/A	☐

6. Presenza al letto del paziente: la possibilità di rimanere accanto al vostro congiunto (il paziente)

Eccellente	☐
Molto buona	☐
Buona	☐
Sufficiente	☐
Scarsa	☐
N/A	☐

7. Se volete aggiungere ulteriori osservazioni (positive o negative) rispetto al ricovero presso questa terapia intensiva e dalle quali potremmo trarre suggerimento per miglioramenti, cortesemente inseritele nel riquadro sottostante.

PARTE 2: SODDISFAZIONE RISPETTO AL PROCESSO DI INFORMAZIONE E DECISIONE RIGUARDANTE LE CURE DEL PAZIENTE CRITICO

In questa sezione, vorremmo porre alcune domande sulle informazioni che avete ricevuto e su come vi siete sentiti coinvolti nel processo decisionale relativo all'assistenza sanitaria del vostro familiare.

BISOGNO DI INFORMAZIONI

1. **Facilità nell'ottenere informazioni:** disponibilità dei membri dello staff di terapia intensiva a rispondere alle vostre domande

Eccellente	☐
Molto buona	☐
Buona	☐
Sufficiente	☐
Scarsa	☐
N/A	☐

2. **Comprensione dell'informazione:** disponibilità dei membri dello staff di terapia intensiva a fornirvi spiegazioni comprensibili delle informazioni ricevute

Eccellente	☐
Molto buona	☐
Buona	☐
Sufficiente	☐
Scarsa	☐
N/A	☐

3. **Onestà dell'informazione:** onestà percepita da voi riguardo le informazioni ricevute sulle condizioni cliniche del vostro congiunto

Eccellente	☐
Molto buona	☐
Buona	☐
Sufficiente	☐
Scarsa	☐
N/A	☐

4. **Completezza dell'informazione:**

4.a. Quanto chiaramente i membri dello staff di terapia intensiva vi hanno informato **su cosa sta succedendo** al vostro congiunto (il paziente)

Eccellente	☐
Molto buona	☐
Buona	☐
Sufficiente	☐
Scarsa	☐
N/A	☐

4.b. Quanto chiaramente i membri dello staff di terapia intensiva vi hanno informato **sul perché determinate cose sono state fatte** al vostro familiare

Eccellente	☐
Molto buona	☐
Buona	☐
Sufficiente	☐
Scarsa	☐
N/A	☐

5. **Coerenza dell'informazione:** avete ricevuto informazioni simili tra loro dal medico, dall'infermiere/a etc...

Eccellente	☐
Molto buona	☐
Buona	☐
Sufficiente	☐
Scarsa	☐
N/A	☐

6. **Qualità globale dell'informazione:**

6a. La qualità complessiva dell'informazione ricevuta dai medici

Eccellente	☐
Molto buona	☐
Buona	☐
Sufficiente	☐
Scarsa	☐
N/A	☐

6b. La qualità complessiva dell'informazione ricevuta dagli infermieri

Eccellente	☐
Molto buona	☐
Buona	☐
Sufficiente	☐
Scarsa	☐
N/A	☐

PROCESSO DECISIONALE

Le decisioni prese dai medici in merito a test diagnostici, interventi chirurgici, trattamenti, ecc.

7. **Inclusione nei processi decisionali:** in che misura il personale vi ha coinvolto nei principali processi decisionali

Eccellente	☐	(andate alla domanda 8)
Molto buona	☐	(andate alla domanda 8)
Buona	☐	(andate alla domanda 8)
Sufficiente	☐	(andate alla domanda 7a)
Scarsa	☐	(andate alla domanda 7a)
N/A	☐	(andate alla domanda 10)

7a. **Se avete trovato l'inclusione nei processi decisionali sufficiente o scarsa,** è stato perché:

Siete stati coinvolti troppo?

Non siete stati coinvolti abbastanza?

Altre motivazioni (si prega di specificare)

____

____

____

8. **Supporto durante i processi decisionali:** quanto bene i membri dello staff di terapia intensiva vi hanno supportati quando sono state prese decisioni importanti

Eccellente	☐
Molto buona	☐
Buona	☐
Sufficiente	☐
Scarsa	☐
N/A	☐

9. Quando sono state prese decisioni importanti, avete avuto sufficiente tempo per poter affrontare e chiarire le vostre preoccupazioni e per avere risposte alle vostre domande?

Ho avuto tempo sufficiente	☐
Avrei voluto avere più tempo	☐
Non so	☐
N/A	☐

VALUTAZIONE COMPLESSIVA

10. Valutate l'assistenza complessiva che il vostro familiare ha ricevuto da tutti i medici, gli infermieri e altri operatori sanitari durante la sua permanenza in terapia intensiva.
(Cerchiare il numero corrispondente)

Peggiori cure possibili	0	1	2	3	4	5	6	7	8	9	10	Migliori cure possibili

11. Se volete aggiungere ulteriori osservazioni (positive o negative) rispetto al ricovero presso questa terapia intensiva e dalle quali potremmo trarre suggerimento per miglioramenti, cortesemente inseritele nel riquadro sottostante.

PARTE 3: SODDISFAZIONE PER LA QUALITÀ DELL'ASSISTENZA AI PAZIENTI NEL FINE VITA

In questa sezione, vorremmo porre alcune domande sulle vostre esperienze in termini di qualità dell'assistenza prestata al vostro familiare negli ultimi giorni della sua vita.

1. Quante volte vi è sembrato che il dolore del vostro congiunto fosse sotto controllo?

Sempre ☐
Per la maggior parte del tempo ☐
Per buona parte del tempo ☐
Per qualche tempo ☐
Per poco tempo ☐
Mai ☐
Non so ☐
N/A ☐

2. Il vostro familiare ha ricevuto aiuto da un ventilatore meccanico per respirare?

Si ☐
No (andare alla domanda 3) ☐
Non so (andare alla domanda 3) ☐

2a. Quante volte vi è sembrato che il vostro familiare fosse a proprio agio con il supporto alla respirazione?

Sempre ☐
Per la maggior parte del tempo ☐
Per buona parte del tempo ☐
Per qualche tempo ☐
Per poco tempo ☐
Mai ☐
Non so ☐
N/A ☐

3. Quante volte vi è sembrato che il vostro familiare mantenesse la sua dignità?

Sempre ☐
Per la maggior parte del tempo ☐
Per buona parte del tempo ☐
Per qualche tempo ☐
Per poco tempo ☐
Mai ☐
Non so ☐
N/A ☐

4. Ritenete che il vostro congiunto abbia ricevuto il sostegno emotivo di cui aveva bisogno?

Si ☐
In parte ☐
No ☐
Non so ☐

5. Ritenete che voi ed i vostri familiari abbiate ricevuto il sostegno emotivo di cui avevate bisogno?

Si ☐
In parte ☐
No ☐
Non so ☐

6. Si prega di valutare l'assistenza complessiva che il vostro familiare ha ricevuto da tutti i medici, infermieri e altri professionisti del settore sanitario durante gli ultimi giorni della sua vita in terapia intensiva. *(Cerchiare il numero)*

Peggiori cure possibili	0	1	2	3	4	5	6	7	8	9	10	Migliori cure possibili

7. Il suo familiare ha discusso le proprie preferenze di trattamento per quanto riguarda cure di fine vita (ad esempio, rianimazione o terapia intensiva) con un medico (medico di famiglia o medico ospedaliero) prima di essere ricoverato in terapia intensiva?

Si ☐
No ☐
Non so ☐

8. Il membro della vostra famiglia ha discusso le sue preferenze di trattamento per quanto riguarda l'assistenza nel fine vita con il personale della terapia intensiva durante il suo ricovero?

Si ☐
Non ne ha avuto possibilità ☐
No ☐
Non so ☐

9. Il trattamento di fine vita è stato portato avanti secondo i desideri del vostro familiare?

Si ☐
Parzialmente ☐
No ☐
Non conoscevo i suoi desideri in merito ☐

10. Ha avuto l'impressione che la vita del suo familiare sia stata prolungata inutilmente?

Si	☐
Parzialmente	☐
No	☐
Non so	☐

11. Ha sentito di aver avuto la possibilità di dire addio al suo familiare?

Si	☐
Parzialmente	☐
No	☐
Non so	☐

12. Se è stata presa la decisione di limitare i trattamenti, era d'accordo su ciò che è stato deciso?

Completamente		☐
Per lo più		☐
Parzialmente		☐
Non molto		☐
Per niente		☐
Non so		☐
N/A	(andare alla domanda 15)	☐

13. Quale parte ha sperimentato in relazione alla decisione di limitare il trattamento di sostegno alla vita?

Il paziente ha preso la decisione	☐
I medici hanno preso la decisione senza coinvolgermi (me o la mia famiglia)	☐
I medici hanno preso la decisione coinvolgendomi (me o la mia famiglia)	☐
La decisione è stata presa unitamente in accordo tra i medici e me (o la mia famiglia)	☐
Io (e/o la mia famiglia) ho preso la decisione dopo essere stato informato della situazione dai medici	☐
Io (e/o la mia famiglia) ho preso la decisione da solo	☐
Non so	☐

In Italia la legislazione stabilisce che le decisioni di limitare le cure a sostegno della vita devono essere prese dal paziente o dai medici. Le famiglie non hanno il diritto o il dovere di prendere decisioni.

14. Se si ignora la legislazione, quale parte si avrebbe voluto avere (lei e/o la sua famiglia) in relazione alla decisione di limitare le cure di sostegno alla vita?

Che i medici avessero preso la decisione senza coinvolgermi (me o la mia famiglia)	☐
Che i medici avessero preso la decisione coinvolgendomi (me o la mia famiglia)	☐
Che la decisione fosse stata presa unitamente in accordo tra i medici e me (o la mia famiglia)	☐
Che io (e/o la mia famiglia) avessi preso la decisione dopo essere stato informato della situazione dai medici	☐
Che io (e/o la mia famiglia) avessi preso la decisione da solo	☐
Non so	☐

15. Se volete aggiungere ulteriori osservazioni (positive o negative) rispetto al ricovero presso questa terapia intensiva e dalle quali potremmo trarre suggerimento per miglioramenti, cortesemente inseritele nel riquadro sottostante.

