# Supplementary material for: Validation and Analysis of the European Quality Questionnaire in Italian Language
Source: Int J Environ Res Public Health. 2020 Nov 28;17(23):8852. doi: 10.3390/ijerph17238852 (PMC7729862; doi:10.3390/ijerph17238852)
Supplement: Supplementary file 1 [file ijerph-17-08852-s001.zip › Supplementary material B Statistical analisys.pdf]

Supplementary material B:

Questionnaire statistical analysis

Methods

In order to evaluate the relationships between the answers to the different questions and to verify whether the domains of the original questionnaire were similar to those obtained with the Italian translation, we used the Multiple Correspondence Analysis (MCA). It is a factorial analysis on qualitative variables built with chi-square metrics. The factorial axes describe in a mutually exclusive way the information contained in the multidimensional space identified by the number of variables considered and by the subjects of the sample. The graphs (of the factorial planes) resulting from the analysis allow to detect the degree of relationship according to the distance between the projected points (variables or subjects). The proportion of explanation of the factorial axis with which each variable contributes (for each of the factorial axes) is an indicator of the importance of the variable in describing the information contained in the set of collected data. Since the coordinates of each subject are a linear combination of the variables used for the construction of the factorial axes, a cluster analysis was performed on them using the Ward algorithm. Similar response profiles were find in each cluster. Five subjects were excluded from the cluster analysis because, due to missing data, they were too different from all other respondents.

Results

This analysis showed that the first two factorial axes explain more than 72% of all the information present in the sample. In the Table 1S we highlighted the questions that were most important in the explanation of the first 3 axes. It also showed how the questions most involved in the explanation of all three axes refer to the quality of the information provided by the medical staff, the explanation of the reason for the maneuvers carried out on the patient and the understanding (effective explanation in relation to the interlocutor) of this information. The most important questions in the explanation of each of the factorial axes are highlighted in bold.

Table 1S. Contribution of the different questions to the explanation of the first three factorial axes.

		%F1	%F2	%F3	F1+F2+F3
	Total explanation	47.6	24.9	8.3	80.8
Acronyms used in the graph	*The Questions*				
A1_*n**	Attention and care by the staff	2.8	2.9	1.7	2.2
A2_a*n*	Pain management	**6.4**	**6.2**	2.8	4.8
A2_b*n*	Management of breathing difficulties	5.9	5.3	1.4	4.3
A2_c*n*	Agitation management	**6.0**	5.4	1.7	4.4
A3_*n*	Atmosphere/intensive care environment	**6.9**	**6.8**	5.5	5.4
A4_*n*	Consideration of family needs	3.9	4.6	3.7	3.3
A5_*n*	Emotional support	5.0	4.8	**7.1**	4.2
A6_*n*	Presence at the patient's bed	**6.3**	5.6	4.6	4.8
B1_*n*	Easy to get information	**6.0**	4.6	7.3	4.6
B2_*n*	Understanding of information	**7.2**	**6.3**	**6.1**	5.5
B3_*n*	Honesty of information	2.7	3.6	**6.3**	2.7
B4_a*n*	How staff give you clear information	4.8	**6.0**	**7.5**	4.4
B4_b*n*	Information on why the manoeuvres were carried out	**6.1**	**6.8**	**9.0**	5.4
B5_*n*	Consistency of information	5.6	4.8	**8.0**	4.5
B6_a*n*	Quality of information provided by medical staff	**6.8**	**9.7**	**9.0**	6.4
B6_b*n*	Quality of information provided by nursing staff	5.8	6.2	**8.4**	5.0
B7_*n*	Inclusion in decision-making processes	4.3	5.2	5.7	3.8
B8_*n*	Support during decision-making processes	4.2	3.4	3.1	3.1
B9_*n*	Time available for decision making	1.2	0.8	0.9	0.8
score10**	Overall assessment of the department	2.6	3.3	1.6	2.2
	TOTAL	100.0	100.0	100.0	

** n is the coding of the answer: 0='no-answer' 1='scarce' 2='sufficient' 3='good' 4='very good' 5='excellent'*

*** this variable has been coded as: 1= ≤8; 2= 9; 3= 10*

The first factorial axis contrast positive and negative judgments on the aspects of information, pain management and agitation. In the second axis there is a qualitative element of the information received and in the third axis the judgements on the understanding of the information provided are differentiated, bringing out the role of the nurse as a language mediator (Figure 1S).

The only item present in the original euroQ2 study, but not very informative in our sample responses, is "Support during decision making".

With the cluster analysis on the coordinates of the factorial axes of each respondent, 4 groups of people that partially define 4 different response profiles were highlighted (Figure 2S).

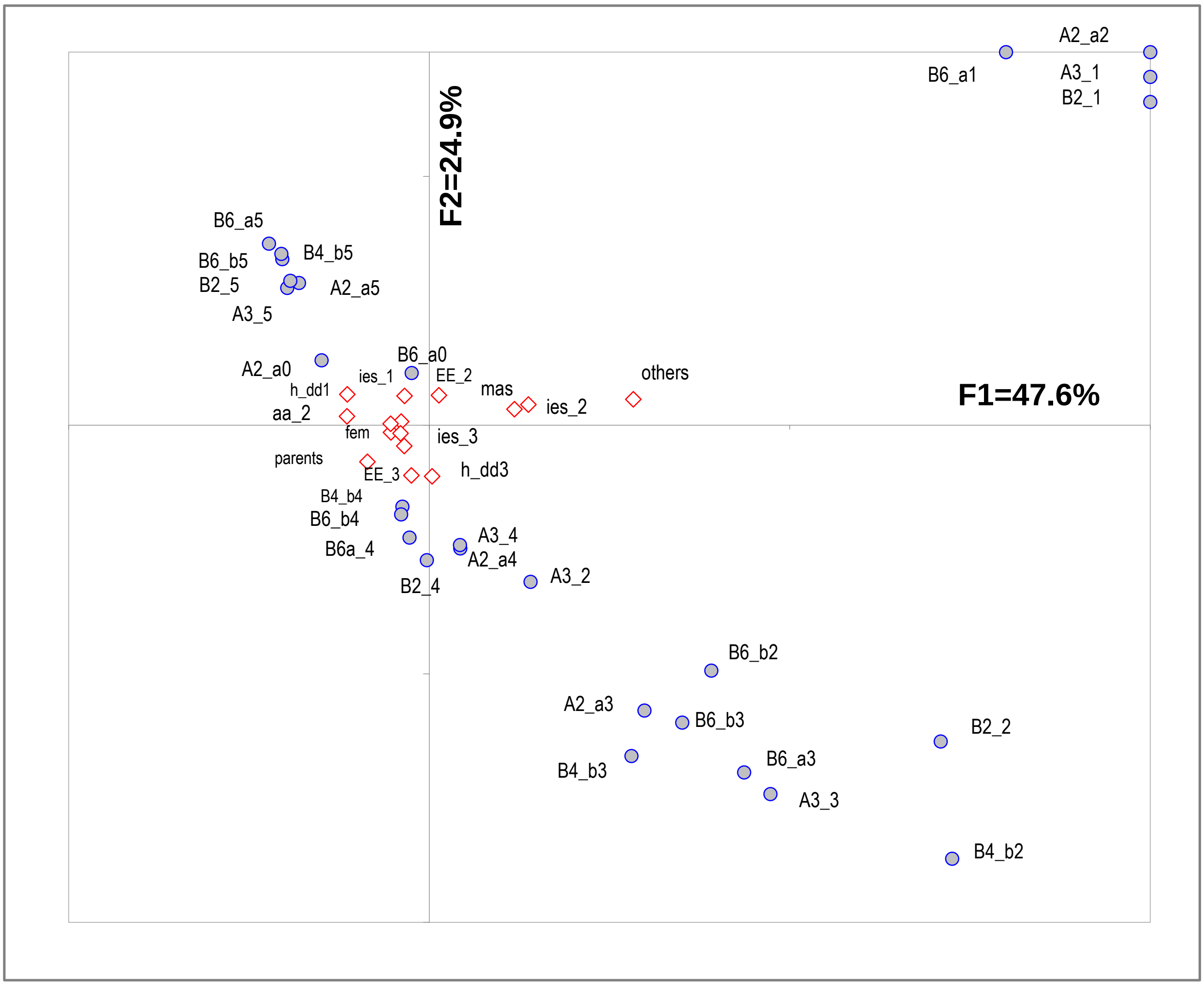

Figure 1S. Multiple correspondence analysis: first factorial plane with the main active variables (circle) and the passive variables (diamond).

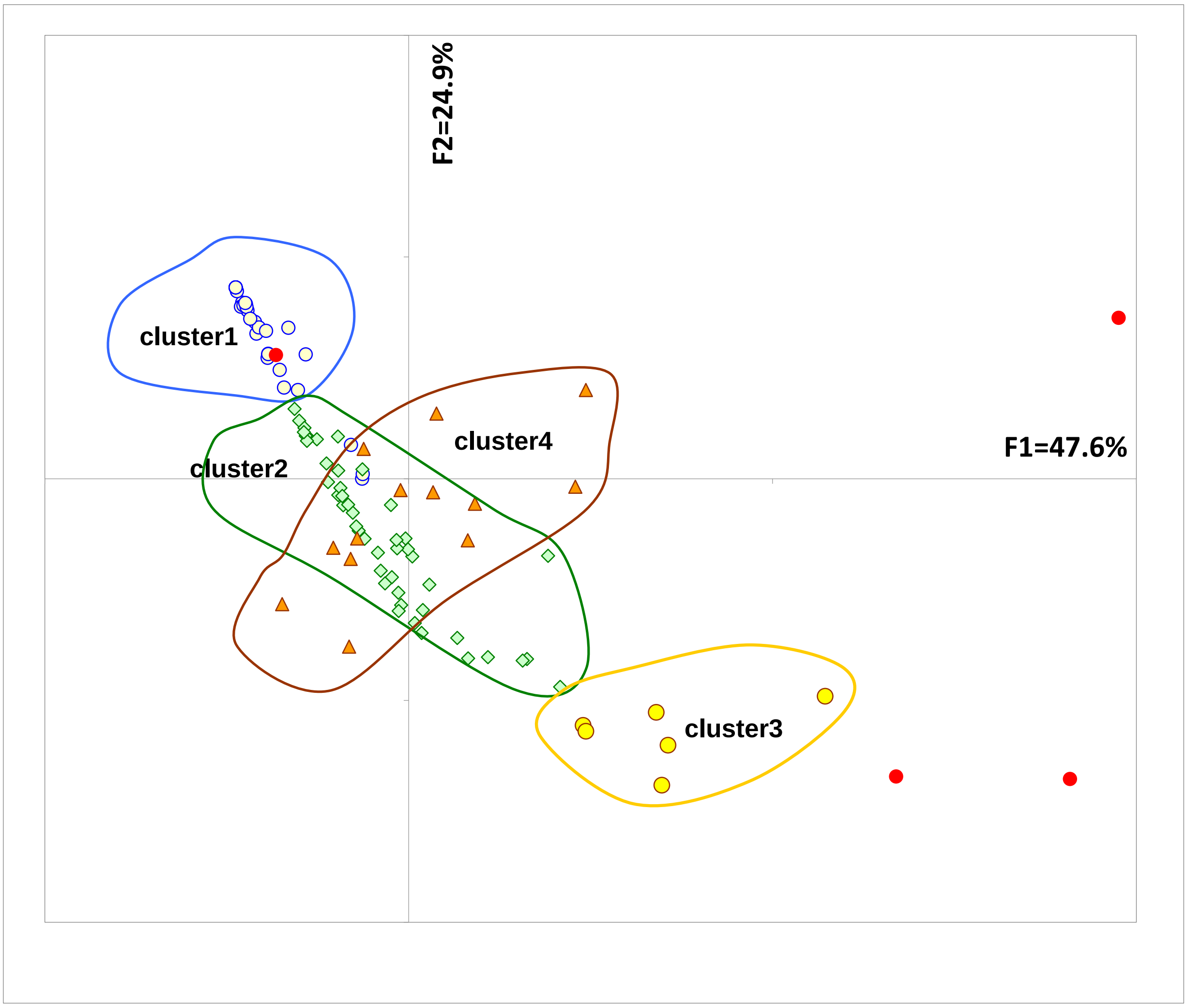

Figure 2S. Cluster analysis: subjects for each cluster with different colours and symbols.

In the first cluster we find a higher proportion of people under 60, males, sons or partners of the patient, who less than others show depression, anxiety and avoidance. They express proportionally more than others a positive judgment on pain management, emotional support received and consideration shown towards family members. In general, they also express very positive judgements about the quality and consistency of the information received and the support for understanding and decision-making.

In the second cluster there is a higher proportion of partners than in others and avoidance attitude is also evident. Proportionally there are fewer sons, a higher proportion of over-60s and anxious people. Again, there is a very positive judgement on the consideration of family members' needs, support and inclusion in decision-making processes and information received.

Cluster 3 is characterized by older people, with little anxiety but a lot of hyperarousal, showing less than others a positive judgement on the care, support and consideration of the needs expressed by the staff. They have a tendency to make less positive judgements about the different aspects investigated.

Cluster 4 shows a higher proportion of women, less than 60 years old, mostly sons, a higher proportion of anxious people and lower proportion than in the others clusters of avoidance and hyperarousal. These people also tend to be less positive about the various questions in the questionnaire, in particular about the support and the degree of inclusion experienced during the decision-making processes.

In Figure 2S the subjects excluded from the clusters are indicated with a red dot because they have a very different profile from all the others (a high percentage of missing answers in particular).

Figure 3S shows a description of the composition of the clusters with reference to all the best answers to the questions considered. Figure 4S shows the difference between the percentage of a certain answer to a question and the percentage of people in a cluster. For example, cluster 1 is composed of the 31.6% of the sample, 27.9% of them are female, therefore in the graph a percentage of -3.7 is reported (Figure 4S). In this way, the specific characteristics of each cluster are showed as more frequent or less frequent.

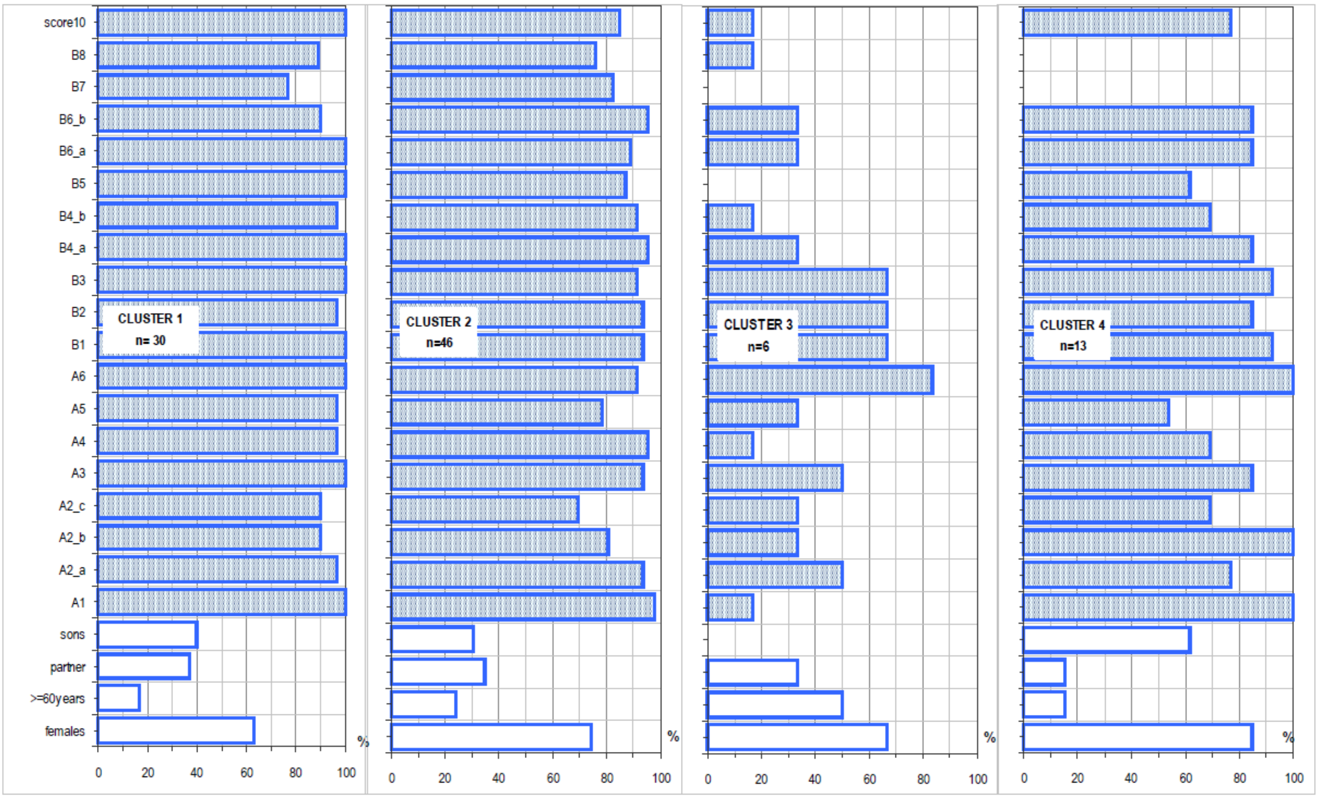

Figure 3S. Distribution (percentage) of the best answers to each question in each cluster. For each euroQ2 question, the best (“excellent” and “very good”) answers are reported.

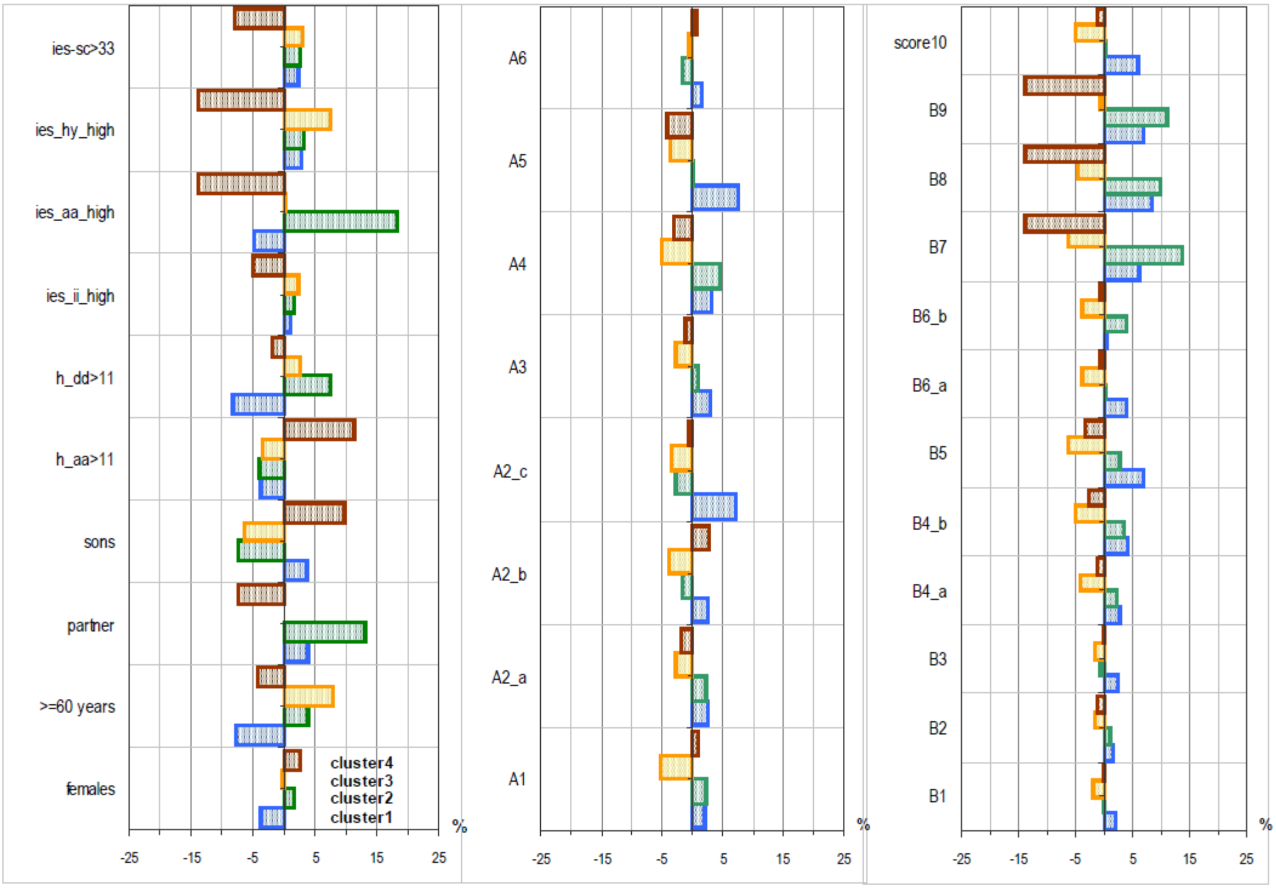

Figure 4S. Difference between the percentage of best answer to a question and the percentage of people in a cluster.

For each euroQ2 question, the best ("excellent" and "very good") answers are reported. h_aa: anxiety from HADS; h_dd: depression form HADS; ies_ii: intrusion from ISE-r; ies_hy: hyperousal from IES-r; ies_aa: avoidance form IES-r.
